# Using a Smartphone App to Identify Clinically Relevant Behavior Trends *via* Symptom Report, Cognition Scores, and Exercise Levels: A Case Series

**DOI:** 10.3389/fpsyt.2019.00652

**Published:** 2019-09-23

**Authors:** Hannah Wisniewski, Philip Henson, John Torous

**Affiliations:** Divison of Digital Psychiatry, Department of Psychiatry, Beth Israel Deaconess Medical Center, Harvard Medical School, Boston, MA, United States

**Keywords:** smartphone, apps, schizophrenia, digital health, psychiatry, digital phenotyping, mental health

## Abstract

The use of smartphone apps for research and clinical care in mental health has become increasingly popular, especially within youth mental health. In particular, digital phenotyping, the monitoring of data streams from a smartphone to identify proxies for functional outcomes like steps, sleep, and sociability, is of interest due to the ability to monitor these multiple relevant indications of clinically symptomatic behavior. However, scientific progress in this field has been slow due to high heterogeneity among smartphone apps and lack of reproducibility. In this paper, we discuss how our division utilized a smartphone app to retrospectively identify clinically relevant behaviors in individuals with psychosis by measuring survey scores (symptom report), games (cognition scores), and step count (exercise levels). Further, we present specific cases of individuals and how the relevance of these data streams varied between them. We found that there was high variability between participants and that each individual’s relevant behavior patterns relied heavily on unique data streams. This suggests that digital phenotyping has high potential to augment clinical care, as it could provide an efficient and individualized mechanism of identifying relevant clinical implications even if population-level models are not yet possible.

## Introduction

Interest in smartphone apps for both research and use in clinical care of psychiatric disorders continues to rise. While there is evidence to support the feasibility of apps across all mental health disease states and all ages, there is a focus on youth mental health because of the high prevalence of ownership and access to smartphones in this demographic. Yet being a “digital native” does not necessitate wanting to use apps and technology towards mental health. Indeed, one recent survey study of college students found that only 26% reported being open to using a mental health app and 81% noted that they would prefer to talk to a person than use an app (1). In this paper, we explore a possible solution to this dichotomy between app and clinicians by discussing case examples of efforts to integrate apps into clinical settings. Focusing on monitoring versus intervention, this paper explores how smartphone apps, digital phenotyping, and real-time mobile data can augment, not replace, face-to-face care for youth.

The potential of smartphones for youth mental health can be understood in part through the concept of digital phenotyping. This refers to “moment-by-moment quantification of the individual-level human phenotype in-situ using data from smartphones and other personal digital devices” (2). In essence, this involves the creation of a digital fingerprint for a user that exhibits the pattern by which they use their mobile device. Common data streams that have been utilized previously in digital phenotyping include geolocation, accelerometer, voice and speech, human–computer interaction, and call/text logs (2, 3). Many of these data streams can serve as proxies for behavior (e.g., accelerometer for activity) or environment (e.g., geolocation data for locations visited) and thus could help provide context to patients’ lived experience of mental illness. For example, in a case series report, Dror Ben-Zeev noted sensor data changes including physical activity, geolocation, phone unlock duration, and speech frequency and duration in participants prior to relapse (4). However, the relevant data streams that indicated a change in clinical status of Ben-Zeev’s participants varied in each of the cases presented, suggesting that the most efficient method of identifying relevant behavior changes may be on an individual level. Thus, we present case reports of our participants based on relevant behavior changes as indicated by a smartphone app.

While this potential of smartphones to improve youth mental health and offer more personalized care is impressive, it is important to note that much of this research and early efforts using smartphones in mental health have not yet be scientifically reproduced. This is in part due to the numerous apps studied, heterogeneity in statistical methods applied to the data, and diversity of patient populations assessed. Given the nascency of digital psychiatry as a field, this current state of affairs is understandable, although the need to now move towards reproducible research is paramount. In that light, the case reports we present here are offered with an online Appendix that includes links to the code repository for others to be able to use the app we used and also the analysis pipelines that generated the below figures.

Another reason for slower progress in digital mental health than may be expected by the prevalence of smartphone and apps existing today is that the optimal use cases for apps in care has not yet been realized. On the one hand, apps may serve as population-level tools capable of screening youth for risk and aiding in prevention. While such population models offer immediate generalizability, there is more evidence to date that these apps may serve as better individual tools designed to assess risk for each person based on their unique situation. But digital tools yielding individual results will require more interpretation, which in turn requires more input and partnerships with clinicians. While many apps today are being built with patient-centered design principles, fewer are created with relationship-centered design in mind—tools to augment the therapeutic alliance between clinicians and patients and advance clinical care. Thus, it is no surprise that a top research priority, as voted by both patients and clinicians, included “What is the optimal way to inclusively implement and combine digital mental health with current clinical care?” (5). While answers to these important questions will take concerted efforts, in this series of case reports, we hope to outline potential directions and next steps.

## Methods

### The Lamp Platform

**Figure 1:**
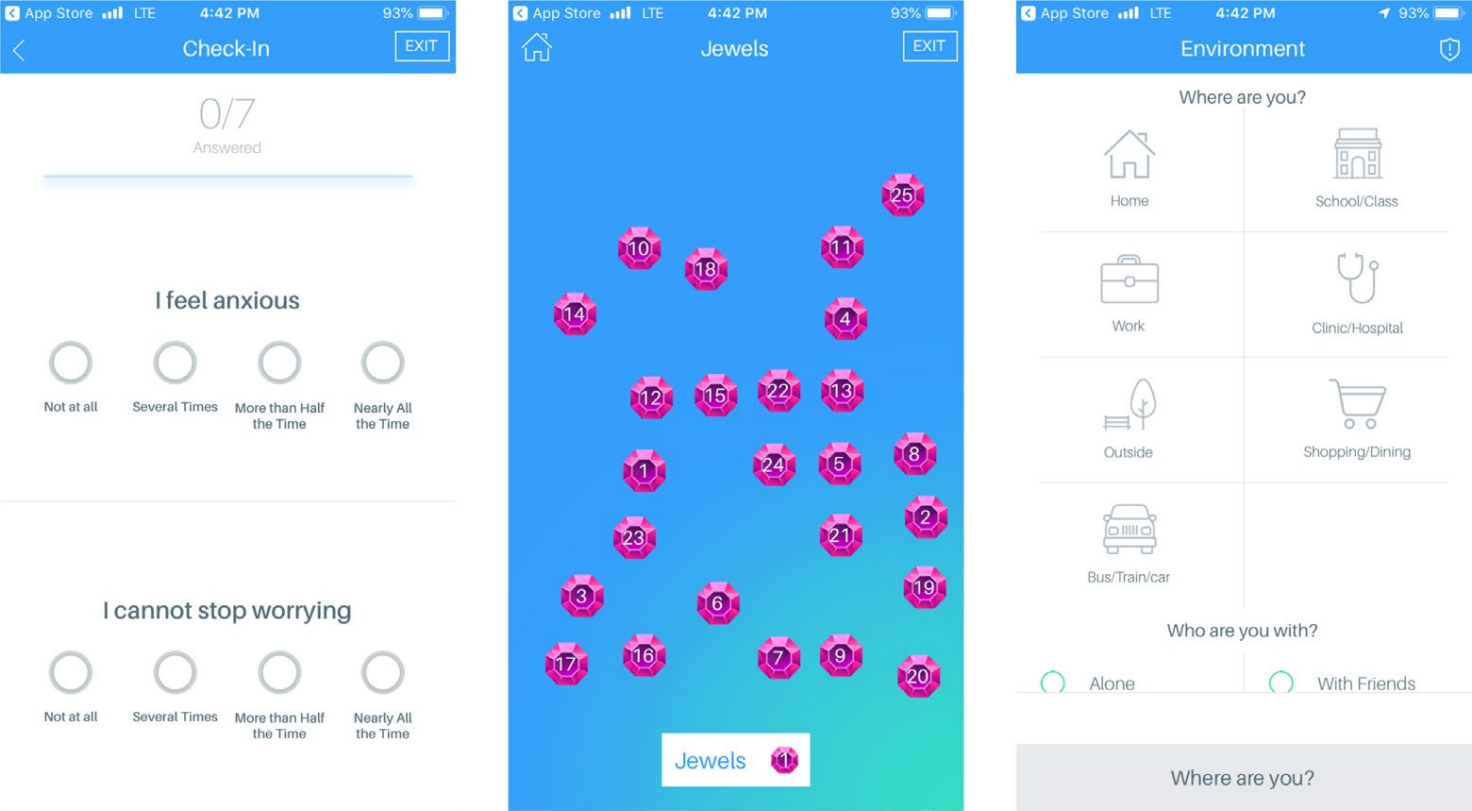
Screenshots of select features of LAMP, including symptom surveys, Jewels A cognitive assessment, and environment and social tagging.

The LAMP app (see **Figure 1**) is a mobile application that can be accessed from both the Apple App store and the Google Play store and is compatible with both Android and iOS operating systems. Currently, to access LAMP, patients must be enrolled in several ongoing research studies across different universities at this time. LAMP collects both active and passive data. Active data include all information collected when the participants are actively using the app (e.g., survey scores, cognitive test scores, and environment and social tagging), whereas passive data include all information collected in the background (GPS location and HealthKit information). LAMP collects several data streams within each of these categories including self-report surveys, cognitive abilities *via* “games” that are modified neuropsychological tests, GPS location, environment and social context tagging, and HealthKit integration from a smartwatch (which includes step count and heart rate).

#### Surveys

LAMP allows the users to take surveys that are customized by clinicians or researchers on the LAMP portal; that is, surveys that have been implemented in the LAMP study aim to assess anxiety, depression, and psychotic symptoms, as well as sociability and sleep quality. LAMP also allows for the customization of survey schedules, which can also be set by the researchers and will send the users a push notification when the researchers want the participants to take a survey. LAMP also records the time taken to answer each question individually, as well as the time taken to complete the entire survey.

#### Cognition Tests

**Figure 2:**
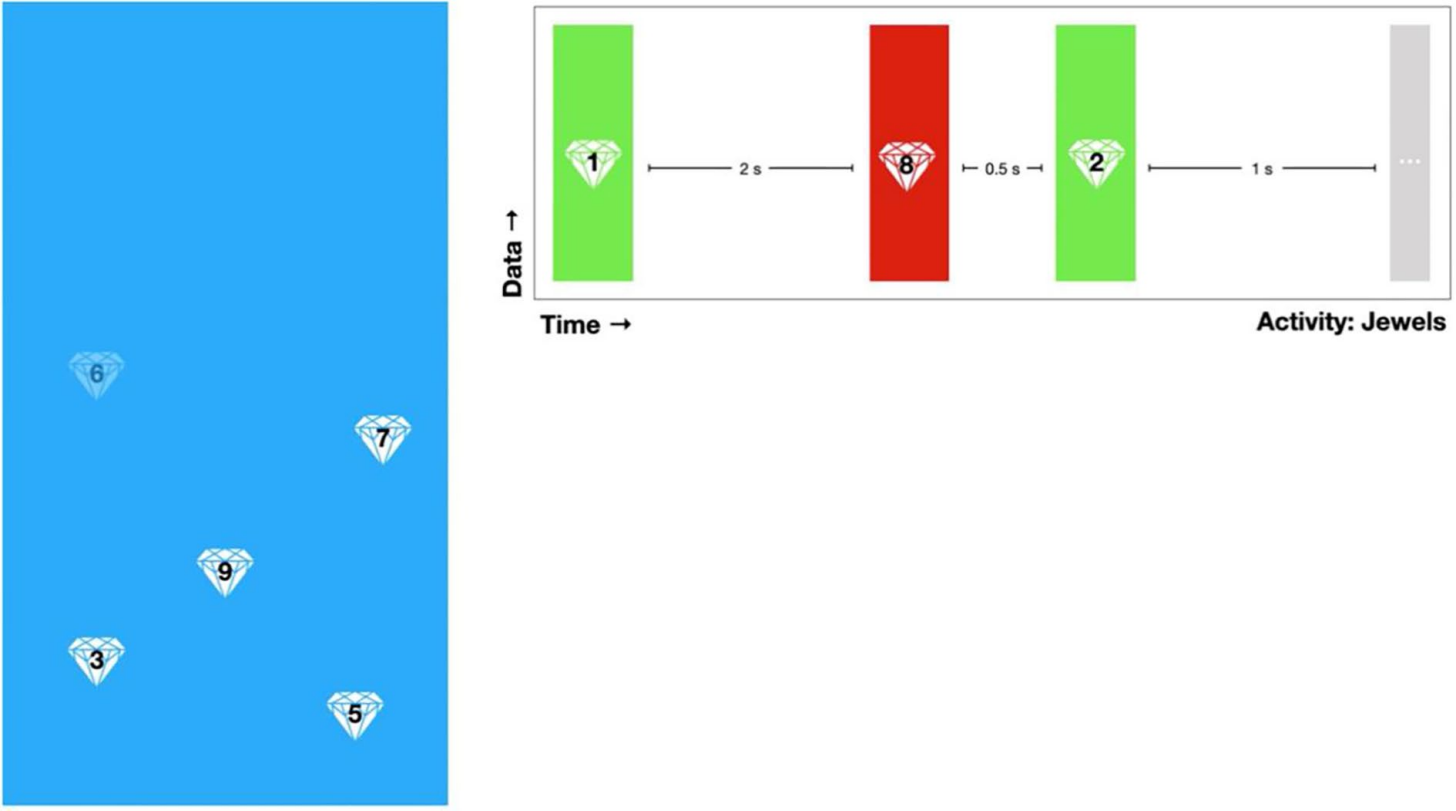
A demonstration of Jewels A. A user is shown numbered jewels on a screen and instructed to tap the jewels in increasing chronological order. The researchers are shown a summary of the user’s taps including time taken and correct/incorrect value.

The LAMP app consists of several “games,” which are simplified neuropsychological cognition tests, to provide a basic assessment of the users’ cognitive abilities. Each cognition test provides the researchers with information specific to that test. For example, Jewels A and B, a modified version of the Trails A and B tests, provides the researchers with the time taken to touch each jewel, the number of correct and incorrect answers, and how long the game took in total. Currently, the majority of the cognition tests assess the users’ memory and attention (see **Figure 2**). A clinician or researcher is able to schedule a notification for a cognition test with the same customization options as surveys. Currently used cognition tests include Jewels A and B, which are modified versions of the trails tasks to assess the users’ attention, and spatial span forwards and backwards to assess the users’ memory.

#### GPS

LAMP collects the users’ GPS location when they complete a game or a survey. In addition, the users are prompted to tag where they are and whom they are with. For example, the users could say they are at home alone. This information allows the researchers to identify any potential trends regarding symptoms/cognition in specific places or with specific people. For example, a participant with agoraphobia may tend to have higher anxiety symptoms while they are at home alone, than in busy public places, which could potentially be useful information to the users in identifying this trend and starting exposure therapy. If the users complete more than one activity in a row, they are not prompted to complete a location tag after every activity, as it is assumed that they are in the same place with the same people. After 30 min, the users are then prompted to tag their location again when completing an activity.

#### Watch and Healthkit Integration

The users are given an Apple Watch or an Android Ticwatch E, depending on their smartphone operating system, to collect exercise and HealthKit data. These include number of steps taken, heart rate, flights of stairs climbed, and miles walked.

#### Dashboard

Researchers or clinicians can access participants’ data through the LAMP Dashboard, which provides visualizations of survey response correlations, average cognition test accuracy, mean cognition test response time, mean survey response time, survey scores, and cognition test standard deviation. The dashboard also provides a summary of each LAMP “event.” A LAMP event marks anytime participants complete a LAMP activity and provide relevant information, including what their scores were on the survey or game, where they took the survey or game, who they were with, and what their HealthKit information was. The dashboard provides a comprehensive summary of the participants’ clinical state.

### Procedure

The study was approved by the Beth Israel Deaconess Medical Center (BIDMC) institutional review board (IRB), and all participants signed written informed consent. Participants come in for an initial study visit, where they first complete informed consent and are informed of all the data streams that LAMP collects. Then, they complete a battery of paper and pencil assessments, including the Patient Health Questionnaire (PHQ-9) (6), Generalized Anxiety Disorder 7-item (GAD-7) (7), Social Functioning Scale (8), the Positive and Negative Symptom Scale (PANSS) (9), and the Brief Assessment of Cognition in Schizophrenia (BACS) (10). Participants are given a smartwatch (either Ticwatch E or Apple Watch Series 3, depending on their operating system) to collect HealthKit information. The patients are fully informed as to how the LAMP app and the smartwatch work at visit 1. After completion of visit 1, the participants may use the app and the smartwatch as much or as little as they would like for 3 months. After 3 months have passed, the participants then return for a second study visit, where they complete the same assessments as they did at visit 1. The data streams examined for this case report included depression, anxiety, psychosis, sleep, sociability, and medication adherence (survey scores), cognitive abilities (Jewels A/B and Spatial Span Forwards/Backwards), and steps (HealthKit). All participants are given an overview of their collected data at visit 2.

During the 3 months between visits 1 and 2, participants are prompted to take surveys 10 times *per* week (twice *per* day, Mondays to Fridays) and prompted to play games five times *per* week (two games at the same time, once *per* day, Mondays to Fridays). On Monday, Wednesday, and Friday, participants were asked to complete surveys assessing depression, sleep, and sociability and to play Jewels B and Spatial Span Forwards. On Tuesday and Thursday, participants were asked to complete surveys assessing anxiety, psychosis, and sociability and to play Jewels A and Spatial Span Backwards. Participants were not compensated for using LAMP or engaging with it so as to better understand real-world use of the app.

### Results

**Figure 3:**
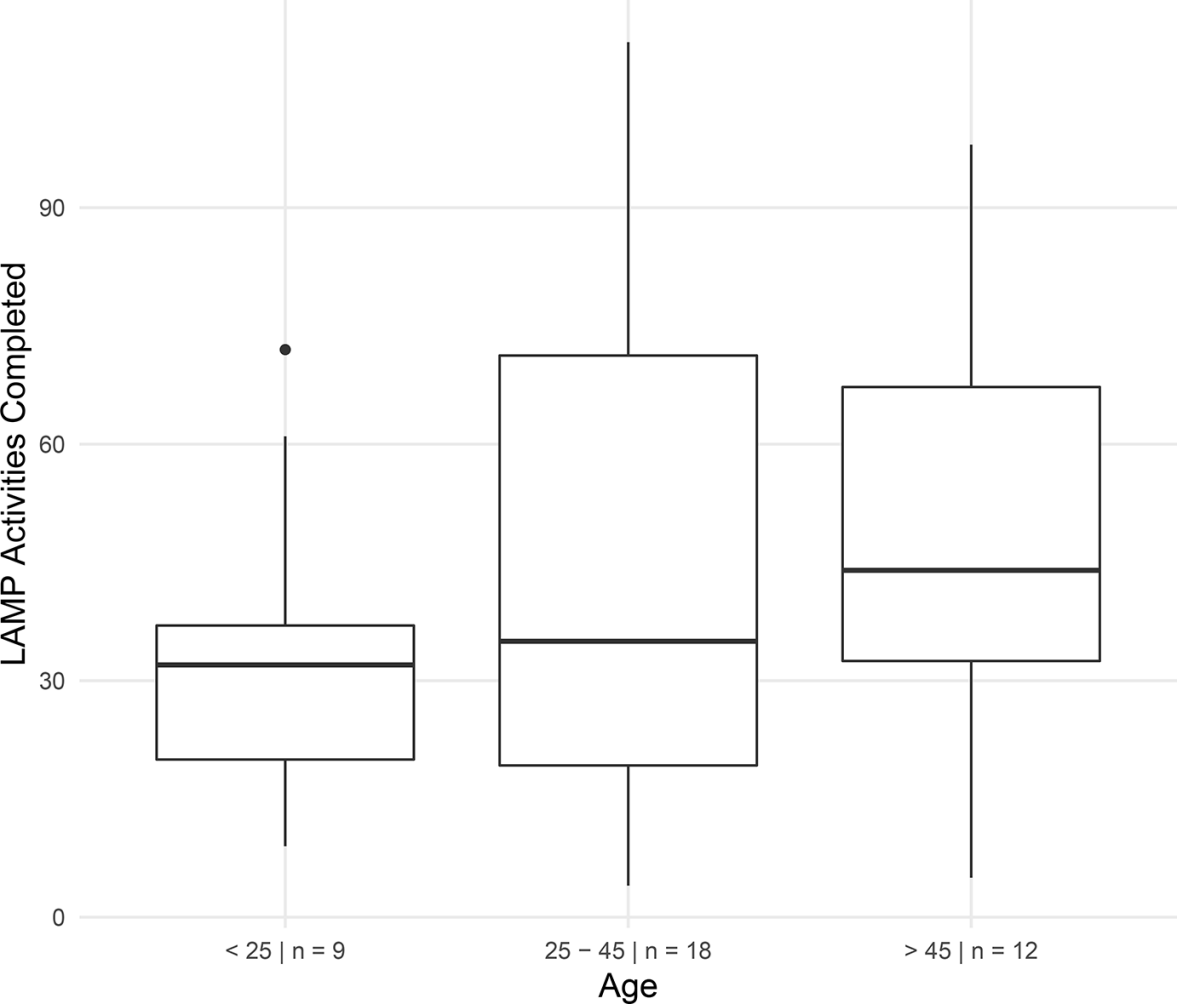
Adherence rate within the first 2 weeks of enrollment in the LAMP study in participants by age. Subjects could complete up to four schedule LAMP activities (surveys and cognitive assessments) per weekday for a total of 20 *per* week and 40 in 2 weeks. Subjects were free to take as many additional tasks as they desired.

Upon examination of overall activities completed by age group within the first 2 weeks of enrollment in the LAMP study, we found that participants under 25 years of age completed less activities (number of games and surveys completed) than did participants above 45 years of age who completed more than the prompts by the app (see **Figure 3**). Participants between 25 and 45 years of age completed approximately the same amount of average activities as those under 25, but with a greater degree of variability.

### Participant 1

Participant 1 demonstrated a high adherence rate with her scheduled surveys in accordance with the scheduled notifications and presents as relatively clinically stable. Thus, Participant 1 is an example of how we are able to map her symptoms and show her overall clinical presentation for monitoring. Interestingly, Participant 1 did not take any sleep surveys within the first month of the LAMP study.

### Participant 2

Participant 2 reported high levels of paranoia, anxiety, and depression and had a relatively stable clinical presentation.

**Figure 4:**
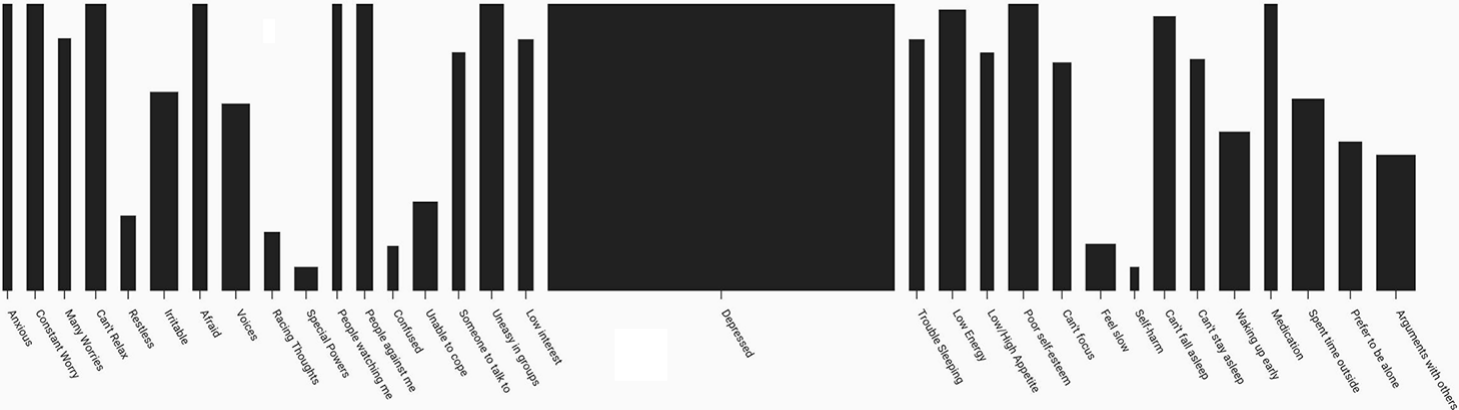
A summary of Participant 2’s average survey scores and time taken to answer survey questions. Height of the bar indicates average survey score, and width of the bar indicates average time taken to respond.

However, when Participant 2 answered survey questions, he, on average, took a longer amount of time answering “Today I feel depressed” compared with any other survey question (see **Figure 4**). In this figure, the height of the bar indicates the answer on a scale of 0–3, and the width of the bar indicates the time taken. This suggests that he continually thought longer about this question when answering it, perhaps indicating uncertainty or high investment in the question. The time taken to complete each question provided by LAMP creates a new data stream that is not accessible when taking these surveys on pencil and paper, underscoring the potential of novel data streams gathered with this method.

**Figure 5:**
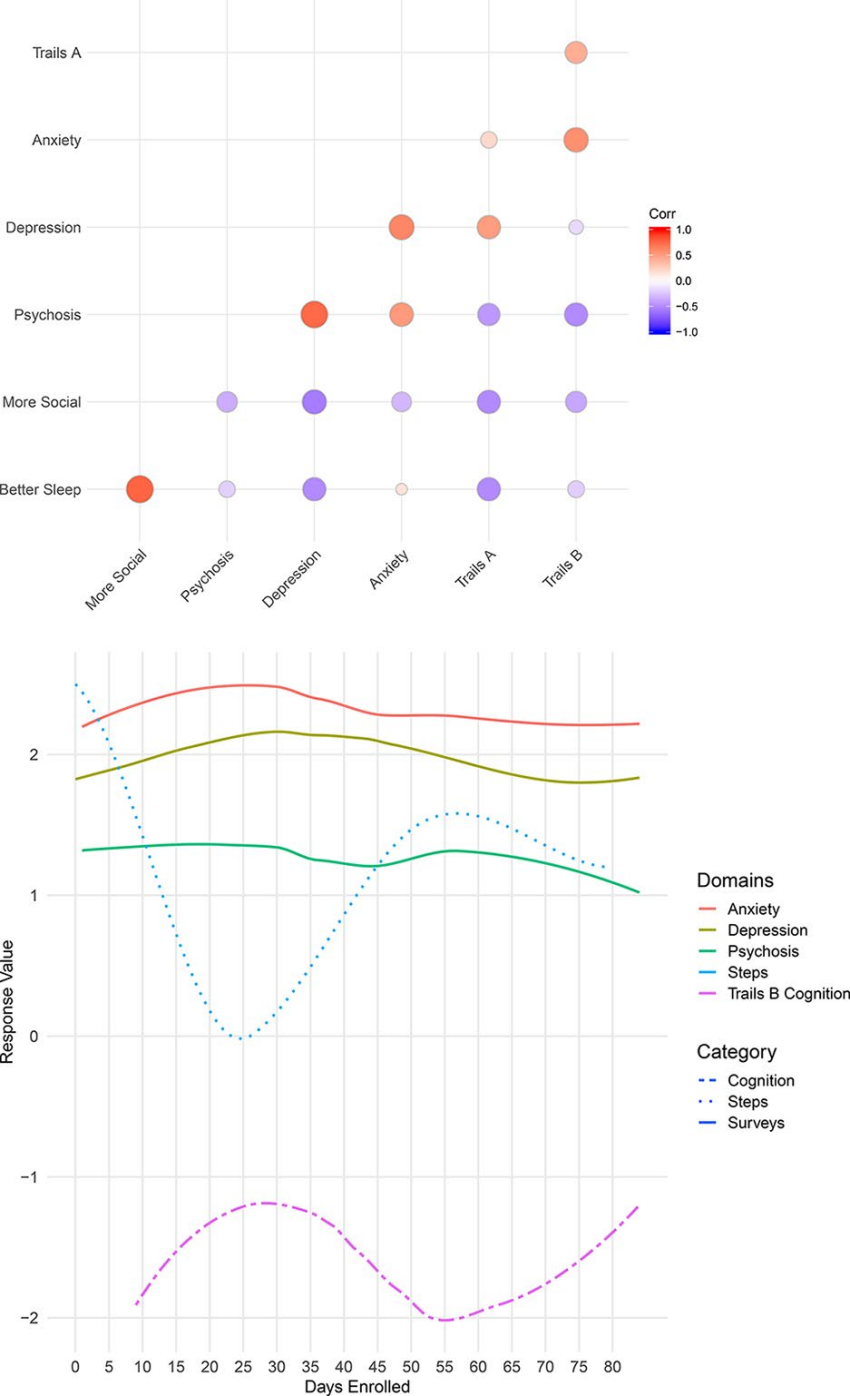
Two views of Participant 2’s data. Correlation between Participant 2’s survey scores and cognition levels (top), and a summary of Participant 2’s anxiety, depression, psychosis, steps, and cognition over time (bottom).

Further, Participant 2 showed strong correlations with his psychotic symptoms in depression, anxiety, steps, and cognition scores (see **Figure 5**). Psychotic symptoms positively correlated with depression and anxiety scores, which is expected and a fairly common presentation among many of the LAMP study participants. Additionally, steps and cognition negatively correlated with psychotic levels, suggesting that when Participant 2 experiences more psychotic symptoms, he exercises less, is less mobile, and has worsened cognition relative to his baseline.

### Participant 3

Participant 3 had been unemployed for 4 months upon enrolling in the study. She reported that has led to a high presence of depressive symptoms (PHQ-9 score of 24). After 2 months of enrollment in the LAMP study, she wished to terminate her participation as she had received a full-time job and would not be able to attend further study visits once her employment started.

**Figure 6:**
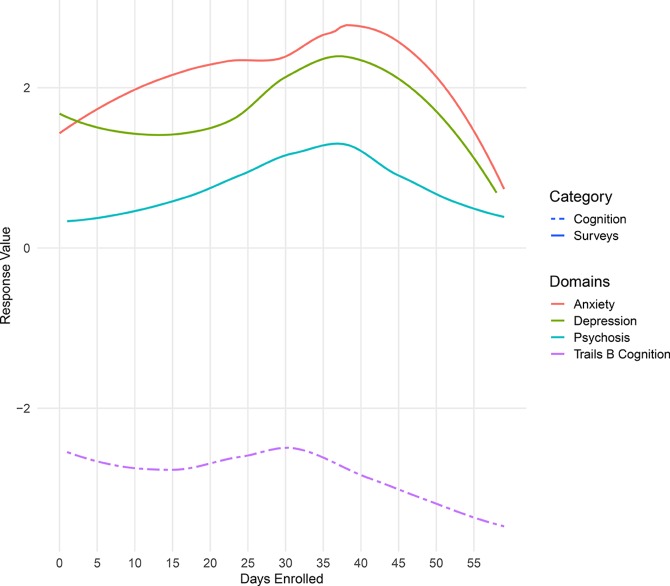
Summary of Participant 3’s survey scores over the duration of her enrollment in the LAMP study.

At her second visit, Participant 3 indicated that her depressive symptoms had improved since receiving a job offer. She denied hospitalization during the duration of her study enrollment. However, upon examination of her survey scores, there was a significant increase in both anxiety and depression scores between days 30 and 45 (see **Figure 6**). Upon inquiry, she indicated that during these days, her symptoms reached what she termed “an all-time high” and discussed hospitalization with her therapist. On approximately day 45, Participant 3 received her job offer. Although her PHQ-9 score was 23 at visit 2, survey scores from LAMP indicated that her symptoms were immediately alleviated upon receiving her job offer. After day 45, her anxiety, depression, and psychosis scores were on average lower than in days 1–30. This provides an example of how digital phenotyping is beneficial in identifying trends for discussion in clinical visits that can help patients share new information that they might have otherwise overlooked.

### Participant 4

**Figure 7:**
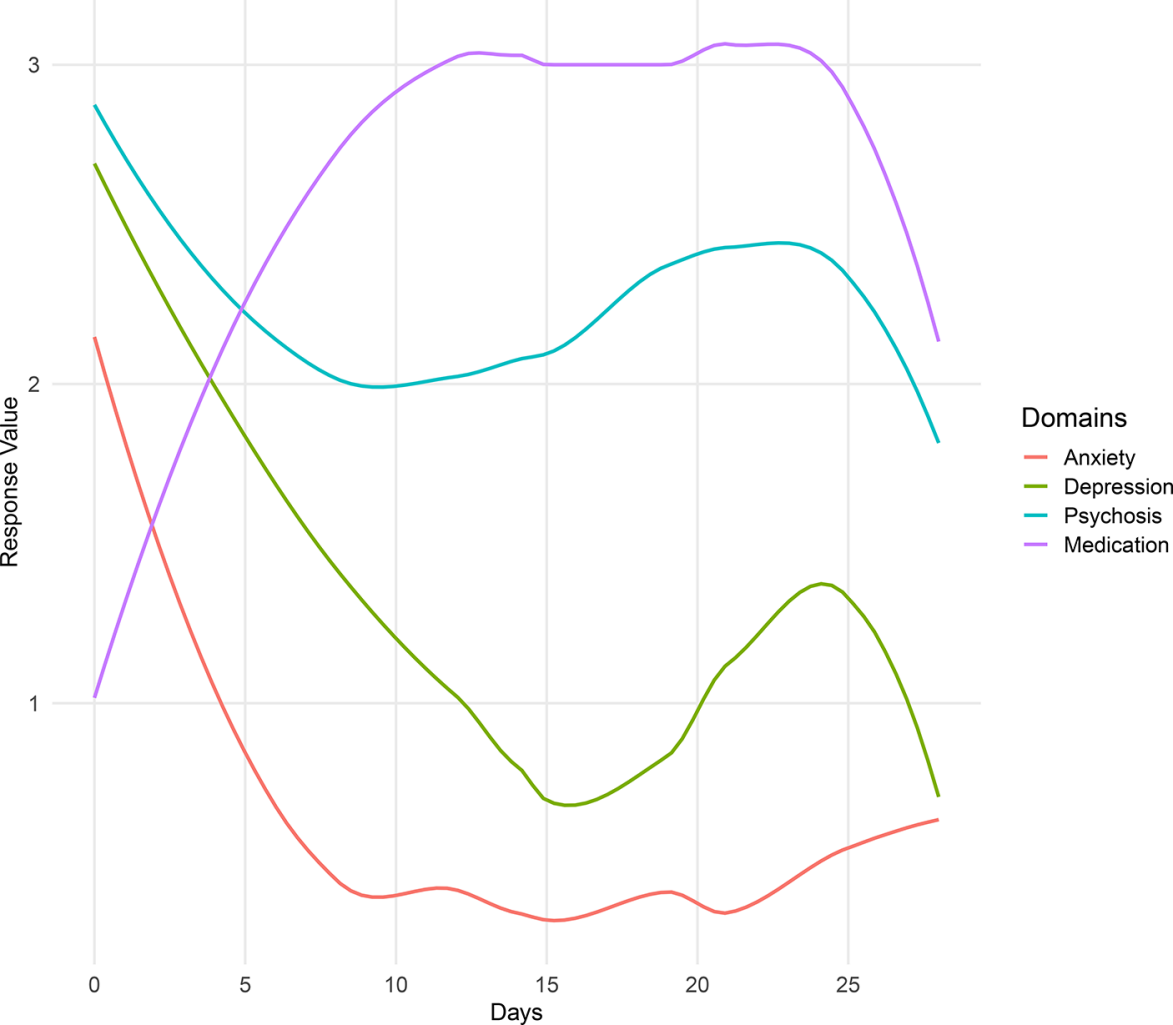
Summary of Participant 4’s survey scores over the duration of her time in the LAMP study.

Participant 4 reported very high anxiety and depression scores, with low medication adherence. However, at approximately 15 days into study enrollment, survey scores for depression, anxiety, and psychotic symptoms began to steadily decrease, while medication adherence increased (see **Figure 7**). As seen in the below figure, we detected changes in symptom report, but we were also able to detect a negative correlation between Participant 4’s medication adherence and her anxiety and depression symptoms.

### Participant 5

**Figure 8:**
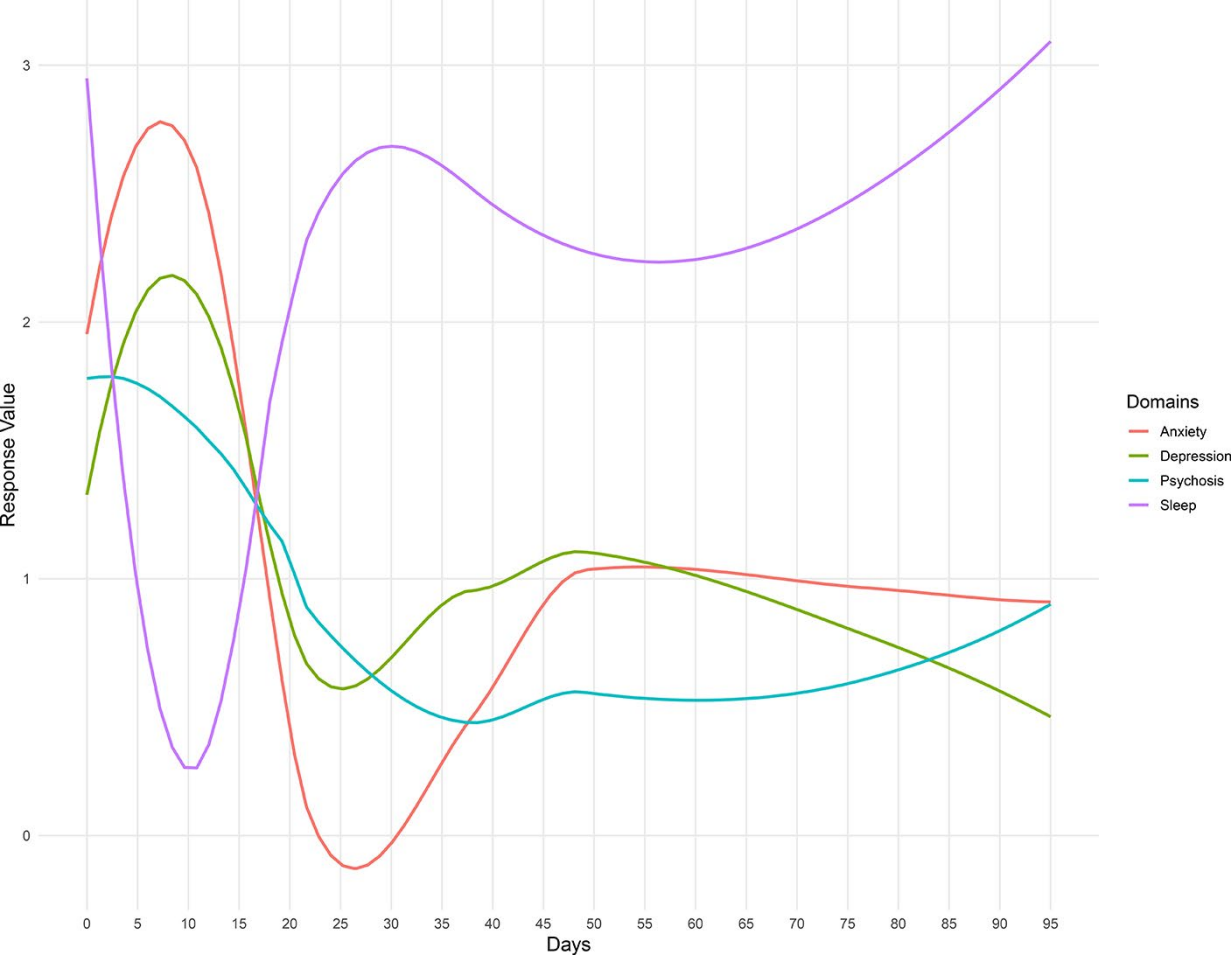
Summary of Participant 5’s survey scores over the duration of her enrollment in the LAMP Study.

Participant 5 reported issues with sleep. Her smartphone data, shown in the **Figure 8**, reveal significant negative correlations between sleep quality and psychosis, anxiety, and depression, suggesting that these symptoms are improved when she sleeps better at night. While correlation does not equal causation, with these data, it is possible to help the patients understand why a trial insomnia cognitive behavioral therapy (CBT) or at least improving sleep hygiene may offer numerous benefits. By using the app, it would also be possible to assess her response to these interventions and help monitor her progress in real time.

## Discussion

In this case report series, we explored diverse clinical use cases around digital phenotyping data for patients with psychotic illness. Utilizing data streams from smartphone-based symptom report, cognition scores, fitness data, and physical and social environment of individuals with psychotic disorders, we investigated behavioral patterns with the goal of identifying potentially clinically relevant and actionable targets for lifestyle and clinical interventions. Our results suggest not only the feasibility of integrating digital phenotyping data in clinical visits but also the potential utility.

Our initial findings provide evidence that use of a smartphone application to monitor symptoms and behaviors produces an individual pattern for each person, indicating that this is a feasible means of monitoring and detecting patient status. Each of the individuals mentioned demonstrated different correlation patterns over time, which provided useful, relevant clinical indications for care. For example, whereas Participant 5’s data showed that her symptoms are strongly correlated with sleep, Participant 4’s data centered around her medication adherence. These correlations indicate that these two participants would benefit from different treatment plans. Because there is a large amount of heterogeneity in presentation among those with psychotic disorders, smartphone usage provides an easy and inexpensive way to monitor these behavior differences.

While identifying individual behavior trends to create personalized treatment plans has high potential to augment clinical care, the high heterogeneity between each of the users provided little ability to establish patterns on a population level. We were unable to find patterns suggesting that certain data streams tended to correlate strongly with others across the larger group of participants. Because of the very individualized nature of the results, further research would be necessary to examine whether or not there is potential for digital phenotyping to be useful on a population level. Additionally, further research is required to create algorithms capable of detecting relevant behavior changes without constant monitoring. This development would allow for less continual monitoring of a patient’s incoming data in a clinical setting and provide an indication for when intervention is necessary based on the detection of clinically relevant behavior.

**Figure 9:**
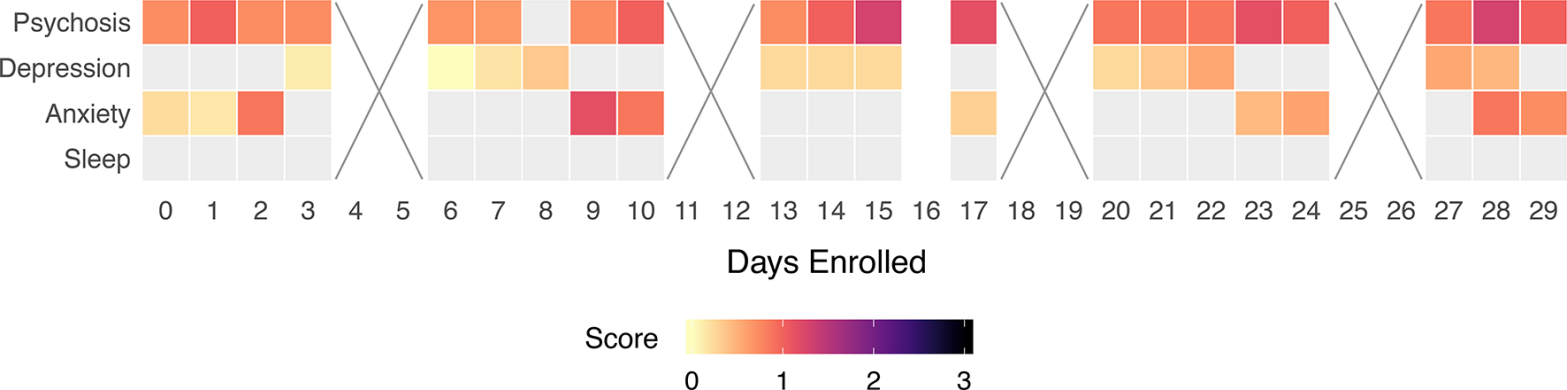
Participant 1’s survey adherence rate within their first 30 days of enrollment. Box color indicates symptom severity. Boxes marked with an “x” indicate a weekend, on which we did not ask participants to complete surveys.

Our results also shed light on the challenge of finding universal use cases and durations of use for mental health app use. While the LAMP smartphone app is able to capture variable patterns among participants that did have clinical relevance, the value in this data was often elucidated though a conversation sparked by the data rather than the raw number or results. For example, Participant 3 had an important clinical finding that the app data suggested but did not reveal without discussing with her. On the other hand, Participant 1 appeared stable, and the data confirmed what was already the current clinical interpretation. Having Participant 1 record symptoms and capture digital data to potentially detect risk of relapse may thus not make sense but could prove useful if there was ever a dramatic change from the stability seen in that data present in **Figure 9**. This becomes a case where patient preference, clinician comfort, and clinical needs will determine use and duration. As seen in all five cases, the potential of LAMP to capture diversity in symptoms also likely requires there be equal flexibility in its clinical use. While our case report series is not able to answer why older participants used the app more than younger participants (**Figure 3**), these results do challenge the popular assumption that digital natives must want digital care.

Although the current study focused on retrospective examination of the data used in discussion with the participants as opposed to continual monitoring and prevention of behaviors, we found that there is potential for digital phenotyping in symptom monitoring and prediction. During discussion, all the users indicated that their observed clinical patterns made sense, and they were able to confirm patterns and stressful life events that warranted their observed change captured by their smartphones. All felt that while the app alone could not yet offer insights into their care, as a tool augmenting care, LAMP was useful.

To make the app more useful, we believe that a new type of mental health provider may be useful to help support its use and interpretation—a digital navigator. While the insights from the app should be discussed in clinical visits, monitoring the data in real time for safety (e.g., risk of suicide) and examining for relevant clinical trends in light of the patient’s unique background and history can be time-consuming without any validated algorithm to help today. Thus, a digital navigator could serve the role of helping the patient set up and learn to use the app, monitoring the data, and highlighting potentially relevant areas for review during clinical visits. This same person could also help with unexpected issues such as the need to reset app passwords, replace smartwatch bands that break, and troubleshoot many other small issues that could arise.

Although our study offers promise that digital phenotyping can capture clinically relevant data, there are several limitations. First, as a case report series, it is difficult to generalize these results, although by making both the app and code to generate plots available, others can seek to replicate this work with their own patient populations. Second, accurate data collection relies heavily on each individual user and how they use their phone (i.e., how often they engage with it and whether or not they carry it wherever they go). Digital phenotyping can only be an accurate proxy for behavior if participants keep their phone with them and use it. Therefore, this method is better suited for individuals who regularly engage with their smartphone and are able to use it with ease. Third, we only included those patients who already owned a smartphone, and so our results are also difficult to generalize to those patients who may use less technology.

## Conclusion

This work demonstrates the feasibility of using smartphone apps to create a digital phenotype in mental health by monitoring symptom surveys, cognition scores, and physiological data. Furthermore, our study has shown that novel data may offer actionable clinical insights on the individual patient level. However, identification of these patterns is only the first step, and we recognize that to use these data in clinical care, there is a need for further replication of results, training of clinicians to use this data meaningfully, and even the creation of new roles for digital navigators to help facilitate the introduction of apps into the clinic.

## Data Availability

The datasets for this study will not be made publicly available because others can use our app and code to generate their own datasets. Those we report on are of unique patients and thus identifiable if the full dataset is offered.

## Ethics Statement

This study is approved by our hospital BIDMC full IRB board.

## Author Contributions

All authors contributed equally to the design, data collection, writing, and editing of the paper.

## Funding

JT is supported by a career development award from the NIMH: 5K23MH116130-02.

## Conflict of Interest Statement

The authors declare that the research was conducted in the absence of any commercial or financial relationships that could be construed as a potential conflict of interest.
